# Change of guard at ACTN

**DOI:** 10.1002/acn3.51721

**Published:** 2023-01-19

**Authors:** Ahmet Hoke

**Affiliations:** ^1^ Johns Hopkins University School of Medicine Baltimore Maryland USA

As I take over the Editor‐in‐Chief position from Dr. Jack Kessler, I want to thank him for his vision for ACTN and 10 years of leadership in setting up the journal and growing it. I have been involved in ACTN from the beginning when Jack asked me to join as an Associate Editor. It has been a privilege to work together to start something new and see it grow. In the beginning, as an open‐access journal, success was not assured, but with Jack's leadership and support from the American Neurological Association and Annals of Neurology, ACTN is now recognized as a reputable and growing journal that publishes impactful research focusing on translational neurology.

I am honored to take the baton from Jack and want to reiterate my commitment to publishing excellent clinical and translational research in neurological sciences. I want to see the ACTN be the home of clinical and translational neurological research that is impactful and timely. With rapid review and publication, ACTN can disseminate new knowledge in a timely manner and affect clinical practice.

As the scientific publication landscape changes, we are seeing a big diversification of the manuscripts submitted to ACTN. The new editorial board and associate editors reflect this diversity. I would like to thank the previous associate editors and editorial board members and welcome the new associate editors, Drs. Jing Ji, Keith Josephs, Samia Khoury, Jun Li, Elisabeth Marsh, Ludy Shih, and Saurabh Sinha. Together, we will strive to have the manuscripts submitted to ACTN reviewed in a timely manner.

During his tenure, Jack Kessler had started InterACTN (InterACTN.org), an interactive case study based on interesting and challenging patients. These online cases and the interactive format proved to be popular with trainees. I would like to thank the current InterACTN editor, Dr. Danny Bega, and welcome the two new coeditors for the InterACTN, Drs. Leana Doherty and Rebecca Tran Pollard. Over the next year, we plan to expand the number of case studies within InterACTN and invite submissions from residents and fellows.

We will be creating a new section to solicit non‐research articles that highlight the humanistic side of being a neurologist. This section will publish short articles and poems about physicians' experiences as doctors. I believe neurology is rich in very rewarding physician–patient experiences and the addition of such a short section would help create a camaraderie among the neurologists. In this section, we also plan to include articles focusing on diversity and social justice and I invite such submissions.

As I close this brief editorial, I again want to thank Jack Kessler and ANA for setting up the journal and invite my colleagues to submit their timely and impactful research and reviews to ACTN.

